# Cheeses Made from Raw and Pasteurized Cow’s Milk Analysed by an Electronic Nose and an Electronic Tongue

**DOI:** 10.3390/s18082415

**Published:** 2018-07-25

**Authors:** Nuno I. P. Valente, Alisa Rudnitskaya, João A. B. P. Oliveira, Elvira M. M. Gaspar, M. Teresa S. R. Gomes

**Affiliations:** 1 CESAM/Department of Chemistry, University of Aveiro, 3810-193 Aveiro, Portugal; nunoipvalente@ua.pt (N.I.P.V.); alisa@ua.pt (A.R.); jabpo@ua.pt (J.A.B.P.O.); 2 LAQV-REQUIMTE, Department of Chemistry, Faculty of Science and Technology, New University of Lisbon, Quinta da Torre, 2825-114 Caparica, Portugal; p123@fct.unl.pt

**Keywords:** cheese, pasteurization, electronic nose, electronic tongue, piezoelectric quartz crystal, acoustic wave sensor, potentiometric sensor

## Abstract

Cheese prepared from whole milk, raw and pasteurized, were analysed by an electronic nose based on piezoelectric quartz crystals and an electronic tongue based on potentiometric sensors, immediately after their preparation and along ripening (after 7 and 21 days). Whey was also analysed by the potentiometric electronic tongue. Results obtained by the electronic nose and tongue were found to be complementary, with the electronic nose being more sensitive to differences in the milk and the electronic tongue being more sensitive to milk pasteurization. Electronic tongue was able to distinguish cheeses made from raw and pasteurized milk, both analysing the whey or the curd, with correct classification rate of 96% and 84%, respectively. Besides, the electronic nose was more sensitive than the electronic tongue to the ripening process, with large differences between samples after 7 and 21 days, while the electronic tongue was only sensitive to the initial maturation stages, with large difference between freshly prepared cheese and with seven days of maturation.

## 1. Introduction

There are several renowned cheeses made from raw milk, in spite of the society pressure towards pasteurization for hygienic reasons. Those concerns were claimed to be in some cases exaggerated due to traditional cheese manufacturing conditions: in very cold regions, under temperatures that prevented microorganisms’ growth. High temperature treatments are also applied in the manufacturing of semi-hard and hard cheeses to inactivate spores of Clostridium tyrobutyricum, in order to prevent late fermentation.

It has been established that heat treatment of milk, especially of cow’s milk, significantly changes its properties, promotes denaturation of whey protein and impairs its rennetability [1,2]. Heating also affects milk salt equilibria, decreasing diffusible calcium and changing the nature of colloidal calcium phosphate [1]. The reversibility of these salt balance modifications depends on the intensity of the thermal treatment.

The question of whether significant alterations on the odour and whey composition are readily detected on the initial ripening stages is the aim of the present investigation. An electronic nose and an electronic tongue were used to analyse cheese and whey produced from raw and pasteurized cow’s milk coagulated with animal rennet. Cheese manufacture, including clotting temperature, was strictly replicated for both raw and pasteurized milk. Calcium was added to both milks, as this is a common practice to restore the milk properties and to ensure coagulation [2]. Besides, calcium is known to influence the coagulation kinetics [3,4,5].

Flavour is one of the main criteria for consumers’ preference and the analysis of cheese bouquet by electronic noses is not new. Sensor arrays based on non-selective metal oxide semiconductor sensors were the most used [6,7,8,9,10], but bulk acoustic wave sensors have also been used [11,12,13]. None of those sensors allowed the identification of individual compounds, but this is not the main objective, as most abundant compounds are often irrelevant in terms of smell. Analytical instruments able to give a complete chemical profile, like GC–MS, are however most often used, in spite of being expensive. Electronic tongues, based on the potentiometric and voltammetric sensors, have been applied to the analysis of fresh and fermented milk [14,15,16,17,18] and milk fermentation monitoring [19]. Reports on the application of the electronic tongue systems to the analysis of cheese are rare [20], probably due to the necessity of the sample preparation, as electronic tongue analyses only liquids.

As both electronic noses and tongues use partially selective or cross-sensitive sensors, pattern recognition and multivariate calibration methods are essential for interpretation of these systems’ outputs. Sample recognition and classification can be performed using a variety of chemometric techniques, the most popular of which are Principal Component Analysis (PCA) [21] and Partial Least Squares–Discriminant Analysis (PLS–DA) [22]. Any classification technique is potentially suitable for the processing of the sensor system data including Support Vector Machines [23], *k*-Nearest Neighbours [24] or Artificial Neural Networks [25], to cite a few examples.

The effort of building such electronic devices, and selecting the sensors, avoiding the presence of redundant sensors of the commercial arrangements, which only function is the input of noise into the system, will compensate in a near future. Portability and the spreading of an affordable analytical methodology may produce significant social changes. Besides the bouquet analysis, the whey analysis by an electronic tongue, gives valuable and complementary information.

## 2. Materials and Methods

### 2.1. Apparatus

#### 2.1.1. Electronic Nose

**Figure 1:**
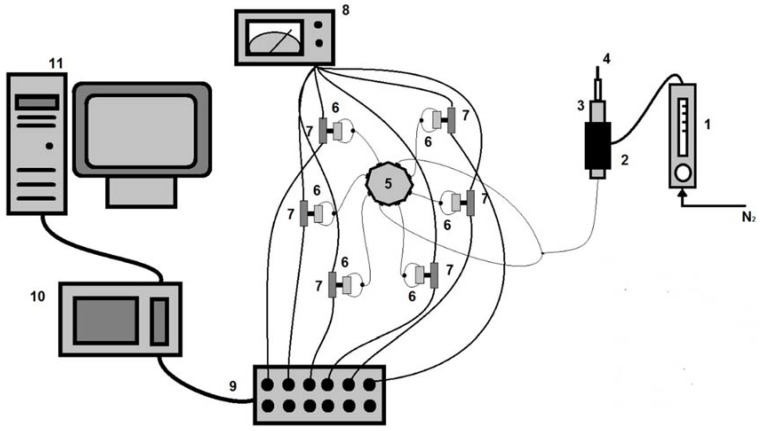
Experimental setup used for the measurements with the electronic nose comprising six bulk acoustic wave sensors. 1: flowmeter, 2: oven, 3: injection port, 4: solid-phase microextraction (SPME) fibre holder, 5: distribution valve, 6: crystal cells, 7: oscillators, 8: power supply, 9: BNC box, 10: Counter/Timer device NI PXI 1033, 11: Computer.

Figure 1 shows the experimental layout. Six sensors, based on coated 9 MHz piezoelectric quartz crystals AT-cut, HC-45 with gold electrodes (Euroquartz, Crewkerne, UK) were used simultaneously. Sensor 1 was coated with carbowax (Supelco 21032, Bellefonte, PA, USA), sensor 2 was coated with polyvinylpyrrolidone (Fluka 81420, Buchs, Switzerland), sensor 3 with triethanolamine (Merck 8377, Darmstadt, Germany), sensor 4 with nafion 117 solution (Fluka 70160, Buchs, Switzerland), sensor 5 with Zinc(II) 2,3,9,10,16,17,23,24-Octatosylaminophthalocyanine (synthesised [26]), and sensor 6 with 1,10-decanedithiol (TCI D0015, Tokyo, Japan). Coating of the piezoelectric quartz crystals was performed spaying solutions of the pure compounds on both faces of the piezoelectric quartz crystals and letting them dry at ambient temperature for two days.

Each piezoelectric quartz crystal was connected to a home-assembled oscillator, based on the Bruckenstein model [27].

The microextraction fibre used to extract the volatile cheese compounds was introduced into the oven at 230 °C, and the desorbed compounds were carried by a nitrogen stream to a distribution valve. The flow was divided in six, and each stream was afterwards divided in two, and directed to each of the sensors’ face.

The frequencies of oscillation of the sensors were simultaneously monitored and stored on a PC at intervals of 1 s, using a Counter/Timer device PXI 1033, from National Instruments, and software written in LabView.

#### 2.1.2. Electronic Tongue

The electronic tongue comprised 11 potentiometric chemical sensors. Eight sensors with plasticized PVC membranes with cross-sensitivity to inorganic and organic anions and cations, a redox sensitive sensor with chalcogenide glass membrane, a pH glass electrode and a crystalline chloride-selective electrode were used. Responses of the sensors were measured vs. conventional Ag/AgCl (KCl 3 M) reference electrode. All sensors used in this study, except the reference and pH electrodes, were produced at the Laboratory of Chemical Sensors of St. Petersburg University [28]. Reference and pH electrode were from Metrohm, Herisau, Switzerland.

Potentiometric measurements were carried out using a custom-made high input impedance multichannel voltmeter connected to a PC.

### 2.2. Milk and Rennet

Whole cow’s milk has been obtained from a local producer immediately after milking and it was used on the milking day. There was no control on animal feeding nor on the animals in the farm that produced the milk along the period this study was carried out. Half of the milk was used without any treatment (R) and the other half was pasteurized by heating at 65 °C for 30 min (P). Animal rennet (HERCO) was bought at a local pharmacy.

### 2.3. Cheese Preparation

A beaker with 40.0 mL of milk was left in a water bath at 32 °C for 1 h, after which calcium chloride (0.1 g L^−1^) and the rennet (0.56 g L^−1^) were added. Mixture was homogenized. Sixteen hours later, the coagulated milk was cut. One hour later, it was drained on a sieve and left at 32 °C for 2 h.

The whey was collected and frozen for analysis by the electronic tongue. Cheese curd was analysed using both electronic nose and tongue.

Curd samples were weighted (2.0 g) and sealed in a 17.0 mL vial together with a magnetic bar. Samples were labelled with a first letter R or P, whether they were prepared with raw or pasteurized milk, followed by four digits, two for the day and two for the month of preparation. Nine vials of each curd sample were prepared: three were analysed by the electronic nose on the day of preparation, three were kept for 7 days at room temperature, and the other three were kept for 21 days at room temperature (24 °C ± 1 °C) before analysis.

Frozen curd and whey samples for electronic tongue analysis were kept at −18 °C and thawed using water bath prior to the analysis.

### 2.4. Analysis

#### 2.4.1. Electronic Nose Measurements

Thirty minutes before the analysis, the cheese vials were immersed in a thermostatic bath at 30 °C. The analysis started with the introduction of the microextraction fibre (75 mm carboxen-polydimethylsiloxane-CAR-PDMS-SPME fibre from Supelco (57318, Bellefonte, PA, USA) in the headspace of the vial, where it was kept for 60 min, while the magnetic bar was spinning. Then, the fibre holder was removed from the vial and the fibre was exposed inside the oven of the analytical system of the electronic nose. Meanwhile, the frequencies of the piezoelectric quartz crystals were stored in a PC at 1 s intervals. The compounds desorbed from the fibre at the high temperature of the oven (230 °C), were carried to the sensors, and their interaction with the sensitive crystal coating produced a frequency decrease proportional to their quantity, and to the sensor sensitivities to those compounds. The analytical response, frequency decrease, was computed for each sensor. Later on, frequencies of all sensors started to increase, until baselines were reached, which was an evidence that the system was ready for a new analysis.

Three vials were analysed by the electronic nose on the same day, and the median of the three responses obtained with each sensor were calculated. Three vials were kept to be analysed 7 days after cheese manufacture and the other three to be analysed at the 21st day. Once again, the median values of the three values were computed.

#### 2.4.2. Electronic Tongue Measurements

Whey samples were diluted 10-fold with ultra-pure water prior to measurements. Concerning curd samples, a weighted amount (0.75 g) was crushed, placed in a cup with 10 mL of ultrapure water and vigorously stirred for 10 min. Resulting liquid was analysed using the electronic tongue. Sensors were washed with ultrapure water until stable potential was reached and then dipped into the sample. Sensors were washed between samples with distilled water for ca. 10 min.

#### 2.4.3. Data Processing

Data processing consisted in the sample recognition and classification. Sample recognition was done using Principal Component Analysis (PCA). Classification models were calculated using Partial Least-Squares regression-Discriminant Analysis (PLS-DA), which is a variant of PLS regression with dummy-dependent variables coding class membership. Two classes were modelled: samples prepared from raw milk and samples prepared from pasteurized milk. Validation was done using cross-validation with 4 segments due to the small number of samples. Unscrambler (version 9.7, CAMO Software AS, Oslo, Norway) was used for PCA and PLS-DA.

#### 2.4.4. Headspace Solid-Phase Micro-Extraction/Gas Chromatography Mass Spectrometry (HS-SPME-GC–MS)

The separations were performed using a fused silica capillary column: SLB-5 ms (Supelco, Bellefonte, PA, USA), 60 m × 0.25 mm I.D., film thickness 0.25 µm. Oven temperature was programmed from 35 °C, hold 5 min, to 240 °C at 4 °C min^−1^, hold 10 min. Helium was used as carrier gas at the flow rate of 1.0 mL min^−1^. High-resolution gas chromatography–mass spectrometry (HRGC–MS) measurements were performed using an Agilent 6850 chromatograph coupled with 5975C MSD with triple-axis detector: mode EI^+^, 70 eV, source temperature 225 °C, interface temperature 280 °C. Full scan mode was used in the range of 40–450 amu, at a rate of 10 scan s^−1^. The injector was used in the splitless mode, at the temperature of 270 °C.

Solid-phase microextraction (SPME) 75 μm carboxen/polydimethylsiloxane (CAR/PDMS) fibre was used in this work for the extraction of volatiles, purchased from Supelco (Bellefonte, PA, USA). Fibre was initially conditioned according to the manufacturer’s instructions: it was inserted into a GC-FID injector during 1 h at 270 °C, in order to remove contaminants and to stabilize the polymeric phase before use. To avoid any background problem, this SPME pre-processing step was applied to all experiments.

Samples (1 g of cheese) were placed into 50 mL EPA (chemically inert clear type I borosilicate glass) vials capped with a PTFE-faced silicone septum. After 1 h, the holder needle was inserted through the septum and SPME fibres were exposed to the sample’s headspace (HS-SPME). Each sample was extracted for 30 min (time to reach the partitioning equilibrium between the sample matrix and the extraction phase) at room temperature. In this case, convection conditions do not affect the amount extracted [29,30]. After extraction, each fibre was withdrawn into the holder needle, removed from the vial, and introduced immediately into the GC–MS injector port for 15 min at 270 °C for thermal desorption of the analytes. Between sampling and sample extraction, a blank was run to avoid carry-over processes.

## 3. Results

**Figure 2:**
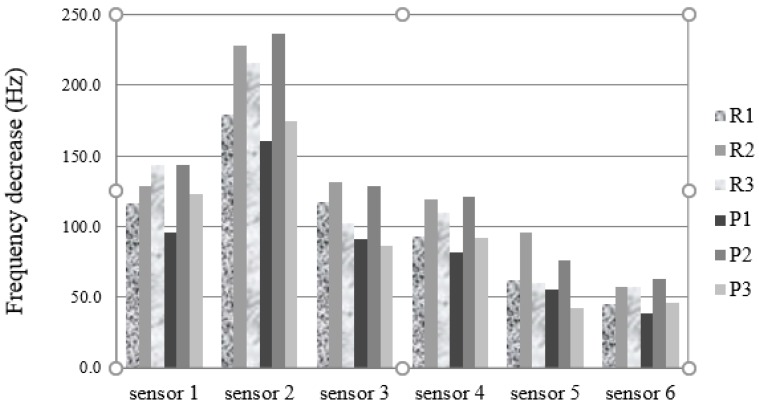
Median of the responses of the array of sensors in the electronic nose to raw (R) and pasteurized (P) cow’s milk cheese, fresh prepared (R1, P1), with 7 (R2, P2) and 21 (R3, P3) days of maturation.

The median of the responses of the electronic nose for the three replicate cheese analysis, of cheeses analysed on the manufacture day, from raw (R1) and pasteurized milk (P1), seven days after its manufacture (R2 and P2) and 21 days after its manufacture (R3, P3), are shown in Figure 2.

For all sensors, the responses on the manufacture day were higher for the raw milk cheese than for the pasteurized milk. Besides, very different ripening patterns can be seen for raw and pasteurized milk cheeses obtained with the six sensors. Looking at the sensor 1 (carbowax sensor), while the responses increase with ripening time for the raw milk cheese, they showed a marked increase from day zero to day seven, and then decreased to the 21st day for the pasteurized cheese. Twenty-one days after manufacture, the responses on all sensors were higher for the raw milk cheese than for the pasteurized milk cheese. For all sensors, and for pasteurized cheese, the responses were much higher one week after cheese manufacture than on the manufacture day, or 21 days after.

The volatile composition of both raw and pasteurized milk cheeses were analysed by GC–MS.

**Figure 3:**
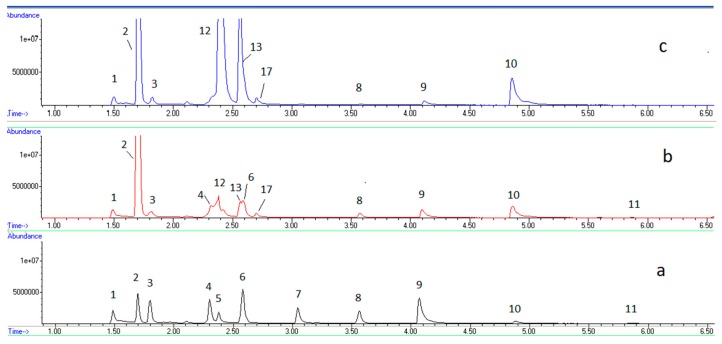
GC–MS chromatograms for cheese prepared from raw milk, along ripening: (**a**) at zero days, (**b**) after 7 days, (**c**) after 21 days.

**Figure 4:**
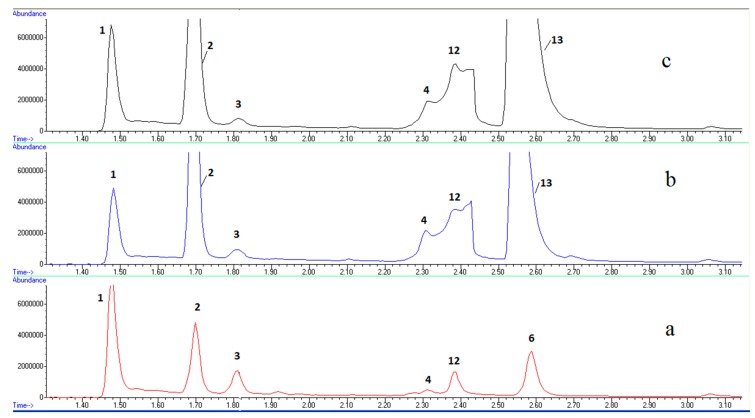

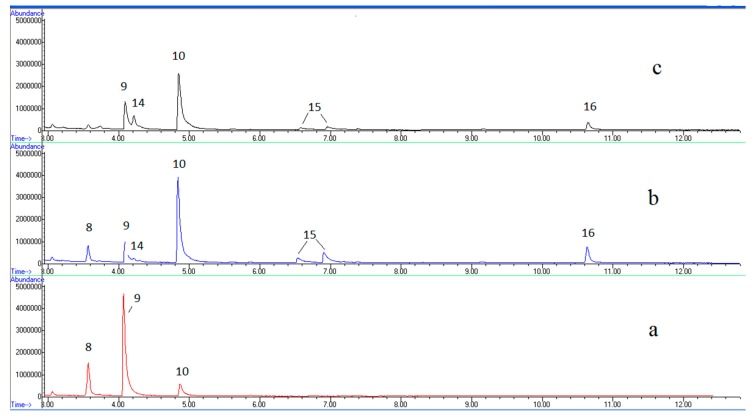
GC–MS chromatograms for cheese prepared from pasteurized milk, along ripening: (**a**) at zero days, (**b**) after 7 days, (**c**) after 21 days.

Main identified compounds are listed on Table 1. Chromatograms of cheese made from raw and pasteurized cheese at several ripening stages can be seen on Figure 3 and Figure 4, respectively. Fresh raw milk cheese showed acetaldehyde, ethanol, acetone, 2,3-butanedione, chloroform, 3-methylbutanal, 3-hydroxybutanone-2 and 3-methylbutanol-1 as dominant volatiles. Fresh pasteurized milk cheese does not contain 3-methylbutanal and it only contains trace quantity of 2,3-butanedione. Maturated pasteurized milk cheese, in addition, contains acetic acid, ethyl acetate, propanoic acid ethyl ester, 2,3-butanediol (the two peaks correspond to two diastereoisomers) and 3-methylbutanol acetate in its volatile composition. Chloroform, 3-hydroxybutanone-2 and 3-methylbutanol-1 disappeared during pasteurized milk cheese maturation.

Sensor 1 responds to many of the cheese bouquet compounds, which makes it impossible to explain this behaviour in terms of chemical changes along ripening. However, the analysis by GC–MS of the compounds adsorbed to the fibre allowed to see that, for instance 3-methylbutanol-1, to which the sensor was responding with a sensitivity of 1.5 Hz/g, experienced an evolution compatible with the observed response trend, i.e., it increased in raw milk cheese from the manufacture day to the first week, and continued to increase until the third week (Figure 3), while in pasteurized milk cheese it showed a maximum 7 days after cheese manufacture, and decreased to the third week (Figure 4).

**Figure 5:**
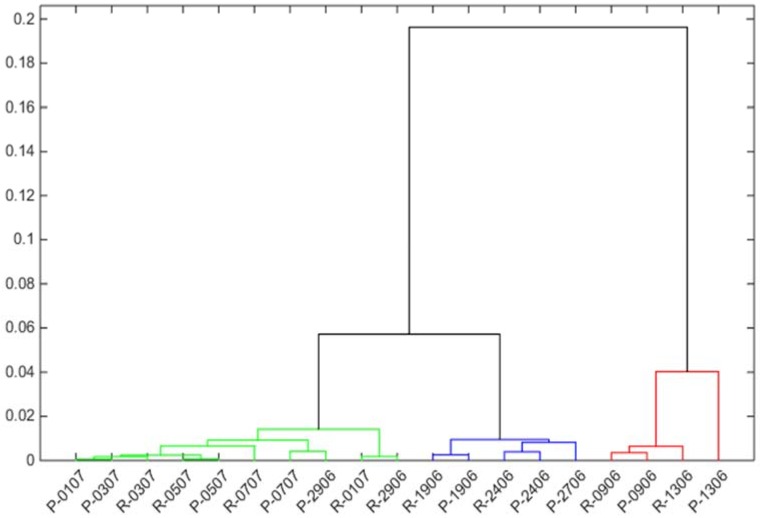
Dendrogram calculated using correlation distance (one minus the sample correlation between points treated as sequences of values) and furthest distance method.

Furthermore, data obtained with the electronic nose with fresh curd from raw and pasteurized milk were evaluated by cluster analysis. Figure 5 shows the dendrogram obtained using correlation distance (one minus the sample correlation between points treated as sequences of values) and furthest distance method.

Observing Figure 5, milk variability is manifest, both in curds produced from raw or pasteurized milk. However, fresh curds produced from the same milk, pasteurized and raw, are situated close to each other, allowing to conclude that variability in the headspace due to the variations in the milk composition from day to day is larger compared to the changes occurring in milk as a result of heating.

**Figure 6:**
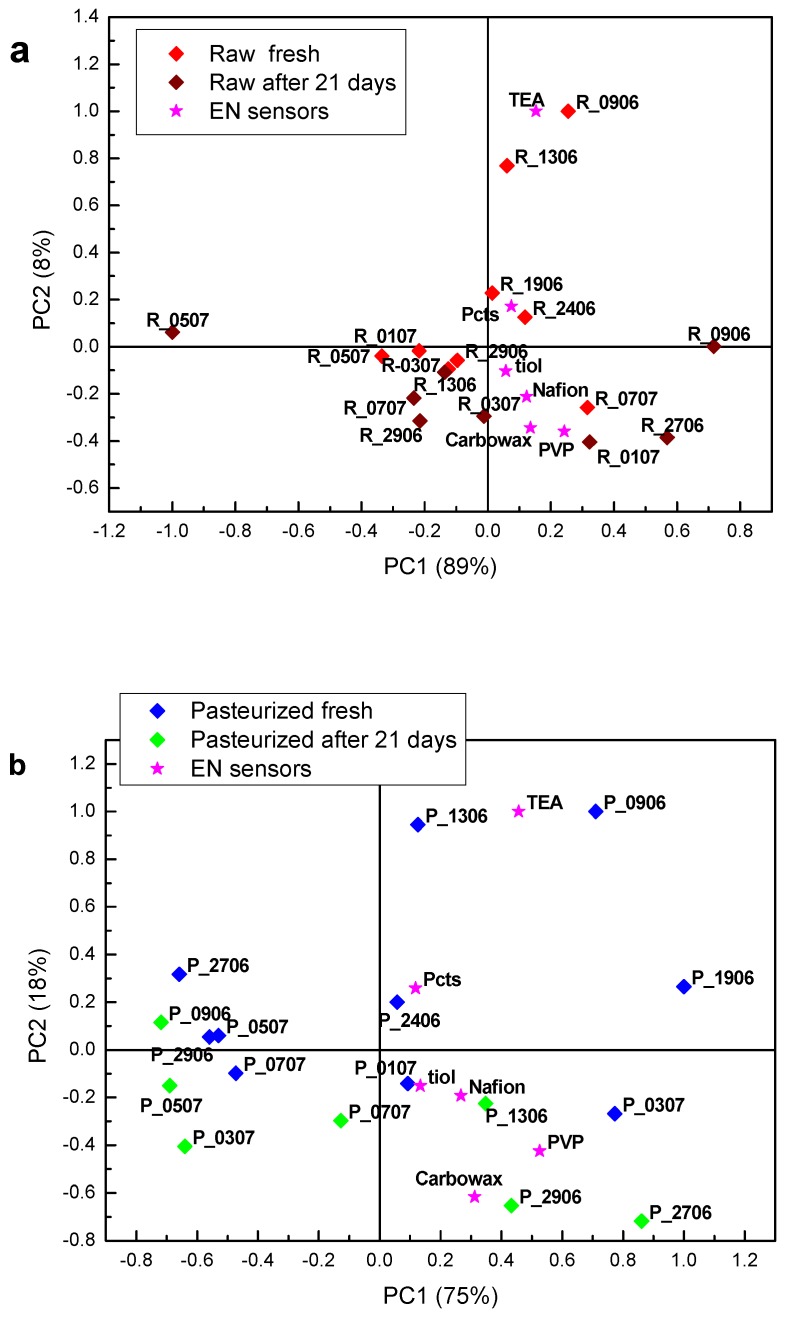
PCA biplot of cheese data, fresh and three weeks matured, obtained from (**a**) raw and (**b**) pasteurized milk by the electronic nose (EN).

Figure 6 shows PCA biplot of cheese data, prepared from raw (Figure 6a) and pasteurized milk (Figure 6b), fresh and three weeks matured, respectively, showing the loading of each variable and the score of each cheese sample, obtained analysing the samples by the electronic nose (EN sensors).

For cheeses produced from raw and pasteurized milk, matured cheeses can be distinguished from fresh samples through PC2 scores. Sensors coated with triethanolamine (TEA), polyvinylpyrrolidone (PVP) and carbowax were the most important ones for this discrimination, with some contribution of Nafion and 1,10-decanedithiol (tiol) coated sensors.

Due to ripening, a few samples, made from raw milk, and the extreme example is R_0507, have moved further away from the cluster, in terms of PC1, and became very different from the rest of the cheese samples. As those samples were prepared from the raw milk, this effect may be explained by the presence of wild microflora, which produces different types/amounts of volatile compounds in the course of the maturing process. As sources of microorganisms in milk may be different, their composition may vary from day to day, which explains why cheeses made from raw milk collected on different days matured differently. This was not observed with the samples prepared from pasteurized milk, which is a confirmation of the potentially richer flavour of the cheeses prepared from raw milk.

**Figure 7:**
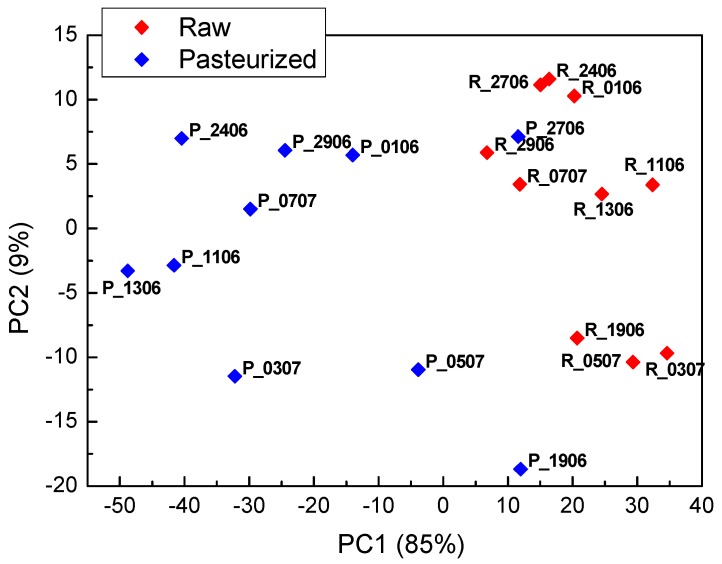
PCA score plot of the measurements of the electronic tongue in the whey collected after cheese preparation using raw and pasteurized milk.

Separation of the whey samples of the cheese produced from the raw and pasteurized milk and measured by the electronic tongue is evident on the PCA score plot of Figure 7.

Only one pasteurized sample (P_2706) is situated in between the set of raw milk samples. Larger dispersion on the score plot of the whey samples obtained from the pasteurized milk compared to the raw milk was observed. Capability of the electronic tongue to discriminate whey from the raw and pasteurized milk was further confirmed by PLS-DA. Classification was correct for all validation samples, except for whey sample P_2706, which was misclassified in the raw milk class, as already noticed from the analysis of the PCA score plot. Classification results are presented in Table 2.

**Figure 8:**
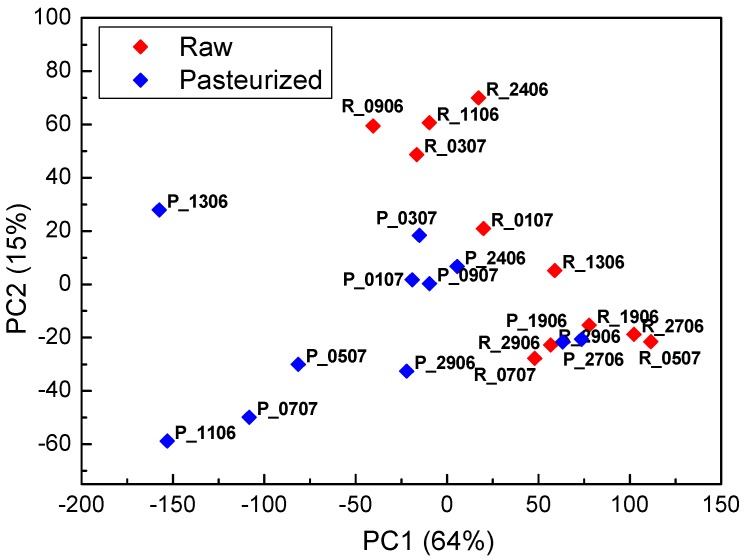
PCA score plot of the measurements of the electronic tongue in the fresh cheese curd samples prepared from the raw and pasteurized milk.

The electronic tongue is expected to respond to different types of compounds from cheese compared to the electronic nose, i.e., mostly to the polar and relatively low molecular weight ones that could be dissolved in the water during sample preparation. PCA score plot of the measurements of the solutions prepared using fresh cheese (Figure 8) shows different sample distribution compared to the one obtained using electronic nose data.

The electronic tongue was able to discriminate between cheese samples made from pasteurized and raw milk, though some dispersion, which can be ascribed to the differences in the composition of milk collected on different days, was observed as well. This result is different from the analysis of headspace made using the electronic nose, indicating that heat treatment affects more significantly dissolved milk compounds than volatiles. In accordance with the results obtained in the whey samples, larger dispersion of the samples made from pasteurized milk was observed, which is probably related to some variations in the pasteurization process itself. Capability of the electronic tongue to distinguish fresh cheeses produced from raw and pasteurized milk was confirmed by classification using PLS-DA. Prediction results were slightly worse compared to the measurements in whey, with three validation samples being misclassified (Table 2).

**Figure 9:**
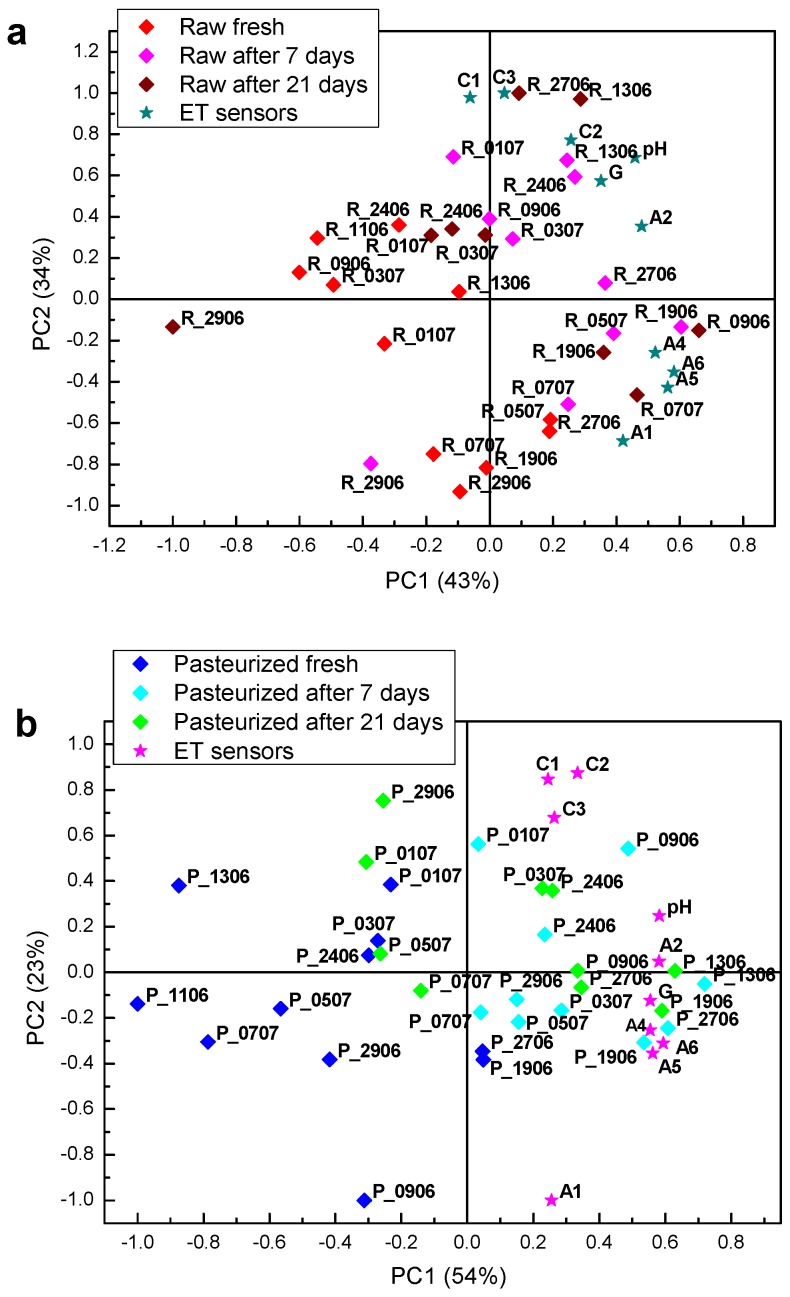
PCA biplot of the measurements of the electronic tongue (ET) in fresh and 7 and 21 days matured cheese samples, prepared from (**a**) raw and (**b**) pasteurized milk.

Evolution of the cheese during maturation is demonstrated in the PCA biplot of the measurements made with the electronic tongue in the solutions of all cheese samples (Figure 9a,b).

Samples of the fresh cheeses are situated at the left part of the score plot, while samples of the cheese matured for 7 and 21 days moved to the right part of the score plot. The same trend was observed for cheeses produced from both raw and pasteurized milk. Large differences were observed between fresh cheese and matured cheese, while cheeses ripened for 7 and 21 days were practically overlapping. This indicates that faster cheese evolution takes place at the beginning of the ripening process or the electronic tongue sensors are more sensitive to the compounds formed at the beginning of the ripening process. Similarly to the electronic nose results, different behaviour was observed for one of the samples prepared from the raw milk, R_2906, which in the process of ripening dislocated in the opposite direction of the other cheese samples. Interestingly, this sample, R_2906, did not stand out in the electronic nose score plot (unfortunately, sample R_0507 matured for 21 days was not available for the electronic tongue analysis). Thus, wild microflora present in the raw milk affects formation of both volatile compounds, i.e., aroma of the cheese, and water-soluble compounds, though these effects depend on the particular microorganism.

**Figure 10:**
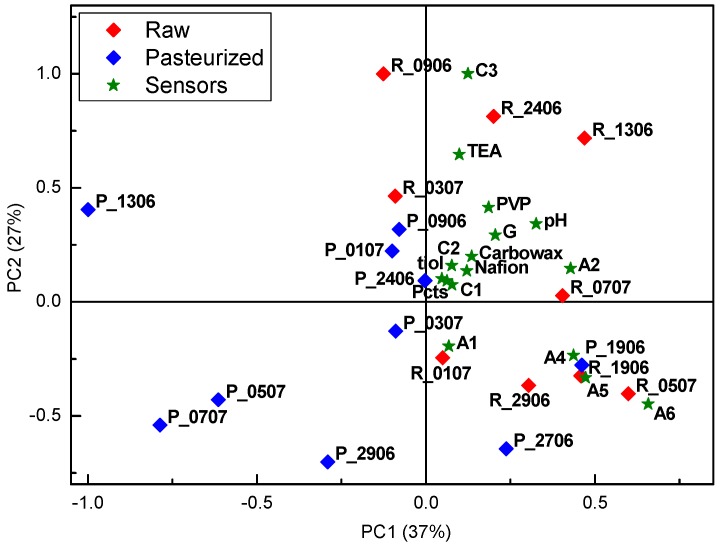
PCA score and loading bi-plot of the fresh cheese samples prepared from raw and pasteurized milk. PCA model was calculated using fused electronic nose and electronic tongue data.

**Figure 11:**
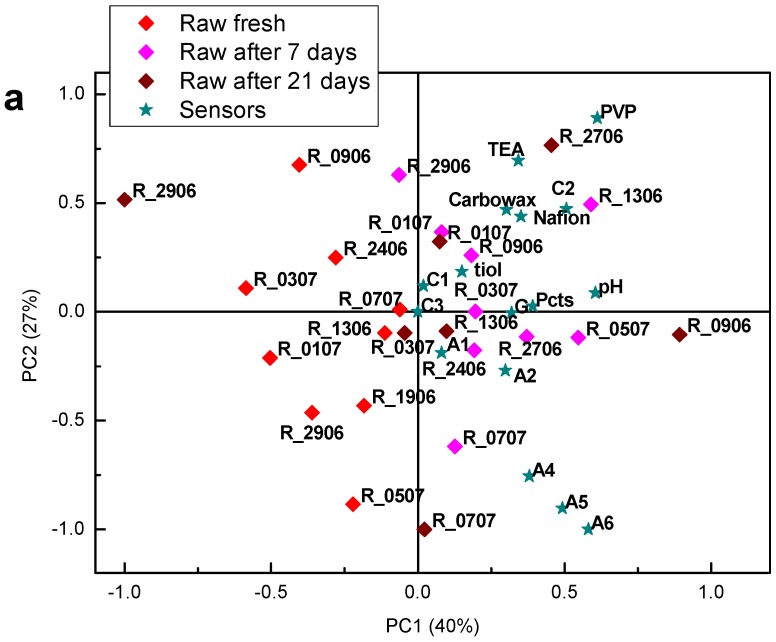

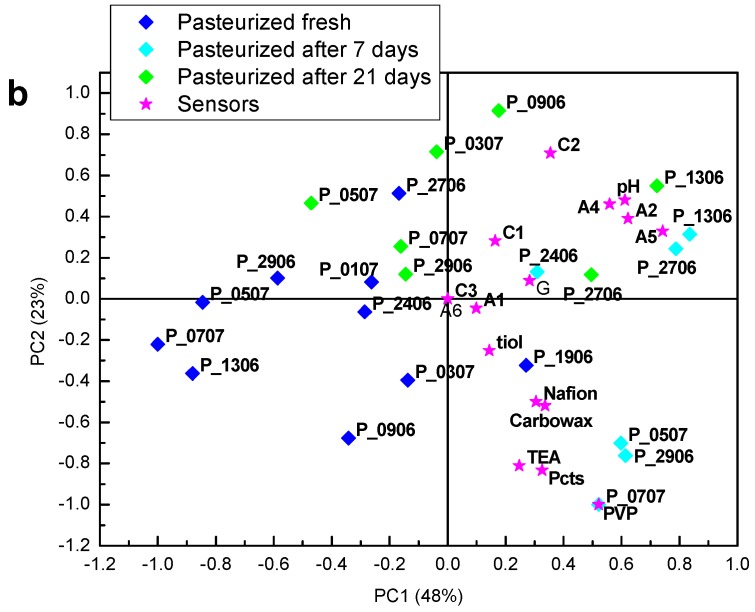
PCA score and loading bi-plot of the fresh and matured cheese samples prepared from raw (**a**) and pasteurized (**b**) milk. PCA model was calculated using fused electronic nose and electronic tongue data.

Finally, fusion of the electronic nose and tongue has been performed with the aim to improve sample discrimination. Approaches to the fusion of the data originating from different instruments that have been described in the literature are categorized as low-, medium- and high-level [31,32,33]. Low-level data fusion, i.e., concatenation of the variables from different origins has been demonstrated to be the most effective when the number of variables in data sets is comparable [33]. Thus, low-level data fusion was adopted in this work. PCA was run on two sets of samples: only fresh cheese samples and all samples. Resulting scores and loadings bi-plots are shown in Figure 10 and Figure 11, respectively. It was observed that data fusion did not bring significant advantages to the discrimination capability of the instruments as partial overlapping of the clusters corresponding to the cheeses prepared from the raw/pasteurized milk or fresh/matured cheese has maintained. However, merging of the data evidenced nonlinearity of the cheese maturation process, particularly for the pasteurized cheese samples that moved during maturation process in anticlockwise direction (Figure 11b) with larger distance between clusters corresponding to the fresh and matured for 7 days cheese than between fresh and matured for 21 days cheese. It is important to note that electronic nose and tongue sensors are not correlated, contributing with complementary information.

## 4. Conclusions

The electronic nose based on acoustic piezoelectric quartz sensors and electronic tongue based on potentiometric chemical sensors were applied to the analysis of fresh and matured cheeses manufactured from raw and pasteurized cow’s milk. In addition, whey samples, collected after cheese preparation, were measured using the electronic tongue. Simple sample preparation method of cheese for measurements with the electronic tongue was employed. Measurements in the fresh cheese samples revealed that the electronic nose displayed higher sensitivity to the difference between milk collected on different days than to the raw and pasteurized milk. Discrimination of whey and fresh cheese samples prepared from raw and pasteurized milk was possible using the electronic tongue with correct classification rate of 96% and 86%, respectively. The electronic tongue could follow cheese maturation process during the first week, while the electronic nose was capable of detecting differences in the maturing process (after 21 days) between cheese made from pasteurized and raw milk.

## Figures and Tables

**Table 1:** Compounds’ identification of raw and pasteurized milk cheeses.

Peak Number	Compound
1	Acetaldehyde
2	Ethanol
3	Acetone
4	2,3-Butanedione
5	Hexane
6	Chloroform
7	3-Methylbutanal
8	Branched alkane
9	3-Hydroxybutanone-2
10	3-Methylbutanol-1
11	Toluene
12	Acetic acid
13	Ethyl acetate
14	Propanoic acid ethyl ester
15	2,3-Butanediol
16	3-Methylbutanol acetate
17	2-Methylpropanol-1

**Table 2:** Results of classification of cheese according to the milk they have been made from (raw vs. pasteurized) using the electronic tongue measurements in whey and solutions prepared using fresh cheese curd. Classification was done using PLS-DA. (Results for validation samples are shown).

Data	Whey	Fresh Cheese Curd
Raw correctly classified	12	10
Raw wrongly classified	0	1
Pasteurized correctly classified	11	9
Pasteurized wrongly classified	1	2
Correct classifications, %	96%	84%

## References

[1] Raynal K., Remeuf F. (1998). The effect of heating on physicochemical and renneting properties of milk: A comparison between caprine, ovine and bovine milk. Int. Dairy J..

[2] Schreiber R., Hinrichs J. (2000). Rennet coagulation of heated milk concentrates. Le Lait.

[3] Noël Y. (1989). Comparison des cinétiques de coagulation enzymatique et mixte du lait. Influence du calcium. Le Lait.

[4] Kowalchyk A.W., Olson N.F. (1979). Milk clotting and curd firmness as affected by type of milk-clotting enzyme, calcium chloride concentration, and season of year. J. Dairy Sci..

[5] Daviau C., Famblard M.-H., Pierre A., Goudédranche H., Maubois J.-L. (2000). Rennet coagulation of skim milk and curd drainage: Effect of pH, casein concentration, ionic strength and heat treatment. Le Lait.

[6] Trihaas J., Vognsen L., Nielsen P.V. (2005). Electronic nose: New tool in modelling the ripening of Danish blue cheese. Int. Dairy J..

[7] O’Riordan P.J., Delahunty C.M. (2003). Characterisation of commercial Cheddar cheese flavour. 1: Traditional and electronic nose approach to quality assessment and market classification. Int. Dairy J..

[8] O’Riordan P.J., Delahunty C.M. (2003). Characterisation of commercial Cheddar cheese flavour. 2: Study of Cheddar cheese discrimination by composition, volatile compounds and descriptive flavour assessment. Int. Dairy J..

[9] Cevoli C., Cerretani L., Gori A., Caboni M.F., Toschi T.G., Fabbri A. (2011). Classification of Pecorino cheeses using electronic nose combined with artificial neural network and comparison with GC-MS analysis of volatile compounds. Food Chem..

[10] Haddi Z., Annanouch F., Amari A., Hadoune A., Bouchikhi B. Application of a portable electronic nose device to discriminate and identify cheeses with known percentages of cow’s and goat’s milk. Proceedings of the IEEE Sensors Conference.

[11] Bargon J., Braschoß S., Flörke J., Herrmann U., Klein L., Loergen J.W., Lopez M., Maric S., Parham A.H., Piacenza P. (2003). Determination of the ripening state of Emmental cheese via quartz crystal microbalances. Sens. Actuators B Chem..

[12] Pais V.F., Oliveira J.A.B.P., Gomes M.T.S.R. (2012). An electronic nose based on coated piezoelectric quartz crystals to certify ewes’ cheese and to discriminate between cheese varieties. Sensors.

[13] Gomes M.T.S.R., Rodríguez Méndez M.L. (2016). Electronic nose in dairy products. The Electronic Nose and Tongue in Food Science.

[14] Winquist F., Bjorklund R., Krantz-Rulcker C., Lundstrom I., Ostergren K., Skoglund T. (2005). An electronic tongue in the dairy industry. Sens. Actuators B Chem..

[15] Ciosek P., Brudzewski K., Wroblewski W. (2006). Milk classification by means of an electronic tongue and Support Vector Machine neural network. Meas. Sci. Technol..

[16] Ciosek P., Wroblewski W. (2006). Miniaturized electronic tongue with an integrated reference microelectrode for the recognition of milk samples. Talanta.

[17] Dias L.A., Peres A.M., Veloso A.C.A., Reis F.S., Vilas-Boas M., Machado A.A.S.C. (2009). An electronic tongue taste evaluation: Identification of goat milk adulteration with bovine milk. Sens. Actuators B Chem..

[18] Paixão T.R.L.C., Bertotti M. (2009). Fabrication of disposable voltammetric electronic tongues by using Prussian Blue films electrodeposited onto CD-R gold surfaces and recognition of milk adulteration. Sens. Actuators B Chem..

[19] Esbensen K., Kirsanov D., Legin A., Rudnitskaya A., Mortensen J., Pedersen J., Vognsen L., Makarychev-Mikhailov S., Vlasov Y. (2004). Fermentation monitoring using multisensor systems: Feasibility study of the electronic tongue. Anal. Bioanal. Chem..

[20] Lipkowitz J.B., Ross C.F., Diako C., Smith D.M. (2018). Discriminating aging and protein-to-fat ratio in Cheddar cheese using sensory analysis and a potentiometric electronic tongue. J. Dairy Sci..

[21] Buratti S., Sinelli N., Bertone E., Venturello A., Casiraghi E., Geobaldo F. (2015). Discrimination between washed Arabica, natural Arabica and Robusta coffees by using near infrared spectroscopy, electronic nose and electronic tongue analysis. J. Sci. Food Agric..

[22] Ciosek P., Brzozka Z., Wroblewski W., Martinelli E., Di Natale C., D’Amico A. (2005). Direct and two-stage data analysis procedures based on PCA, PLS-DA and ANN for ISE-based electronic tongue-Effect of supervised feature extraction. Talanta.

[23] Liu M., Wang M., Wang J., Li D. (2013). Comparison of random forest, support vector machine and back propagation neural network for electronic tongue data classification: Application to the recognition of orange beverage and Chinese vinegar. Sens. Actuators B Chem..

[24] Güney S., Atasoy A. (2012). Multiclass classification of n-butanol concentrations with k-nearest neighbor algorithm and support vector machine in an electronic nose. Sens. Actuators B Chem..

[25] Szulczyński B., Armiński K., Namieśnik J., Gębicki J. (2018). Determination of odour interactions in gaseous mixtures using electronic nose methods with artificial neural networks. Sensors.

[26] Farinha A.S.F., Calvete M.J.F., Paz F.A.A., Tomé A.C., Cavaleiro J.A.S., Sessler J.L., Tomé J.P.C. (2014). Octatosylaminophthalocyanine: A reusable chromogenic anion chemosensor. Sens. Actuators B Chem..

[27] Bruckenstein S., Shay M. (1985). Experimental aspects of the use of the quartz crystal microbalance in solution. Electrochim. Acta.

[28] Legin A., Rudnitskaya A., Vlasov Y. (2003). Electronic tongues: New analytical perspective for chemical sensors. Compr. Anal. Chem..

[29] Pawliszyn J. (2000). Theory of Solid-Phase Microextraction. J. Chromatogr. Sci..

[30] Vas G., Vékey K. (2004). Solid-phase microextraction: A powerful sample preparation tool prior to mass spectrometric analysis. J. Mass Spectrom..

[31] Di Rosa A.R., Leone F., Cheli F., Chiofalo V. (2017). Fusion of electronic nose, electronic tongue and computer vision for animal source food authentication and quality assessment—A review. J. Food Eng..

[32] Di Rosa A.R., Leone F., Scattareggia C., Chiofalo V. (2018). Botanical origin identification of Sicilian honeys based on artificial senses and multi-sensor data fusion. Eur. Food Res. Technol..

[33] Rudnitskaya A., Kirsanov D., Legin A., Beullens K., Lammertyn J., Nicolai B.M., Irudayaraj J. (2006). Analysis of apples varieties—Comparison of electronic tongue with different analytical techniques. Sens. Actuators B Chem..

